# Supplementary material for: Engineering the Electronic Structure towards Visible Lights Photocatalysis of CaTiO3 Perovskites by Cation (La/Ce)-Anion (N/S) Co-Doping: A First-Principles Study
Source: Molecules. 2023 Oct 17;28(20):7134. doi: 10.3390/molecules28207134 (PMC10608951; doi:10.3390/molecules28207134)
Supplement: Supplementary file 1 [file molecules-28-07134-s001.zip › molecules-2651789-supplementary.pdf]

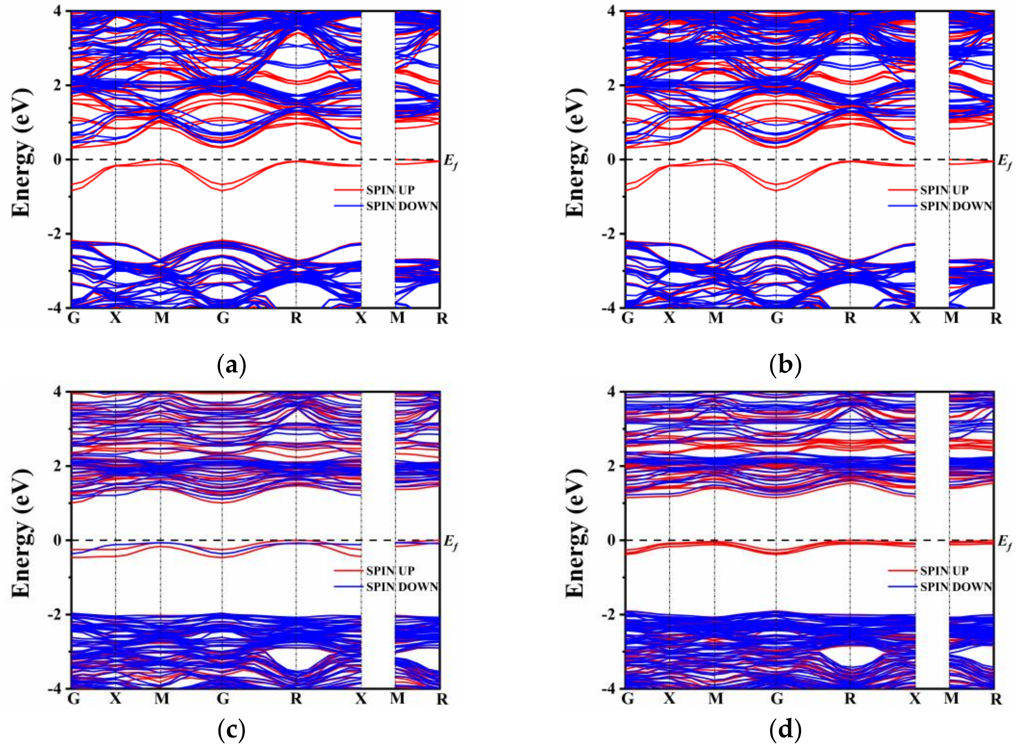

Figure S1. The band structures of (a) LCTO-0.25, (b) CCTO-0.25, (c) LCTO-0.375, and (d) CCTO-0.375.

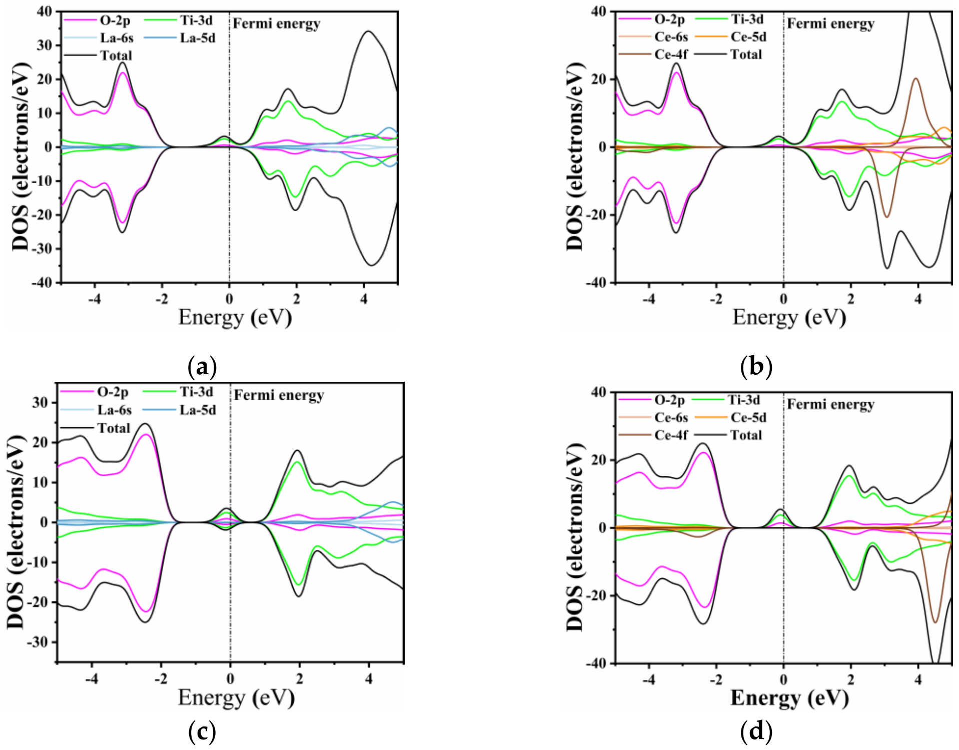

Figure S2. The TDOS and PDOS of (a) LCTO-0.25, (b) CCTO-0.25, (c) LCTO-0.375, and (d)CCTO-0.375.

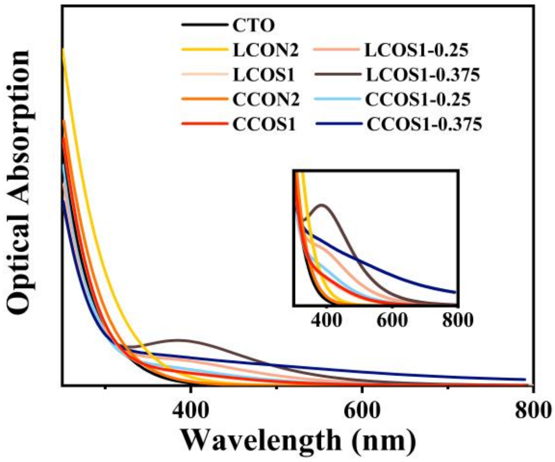

Figure S3. Optical absorptions of pure and (La/Ce-N/S) Co-doped CTO.

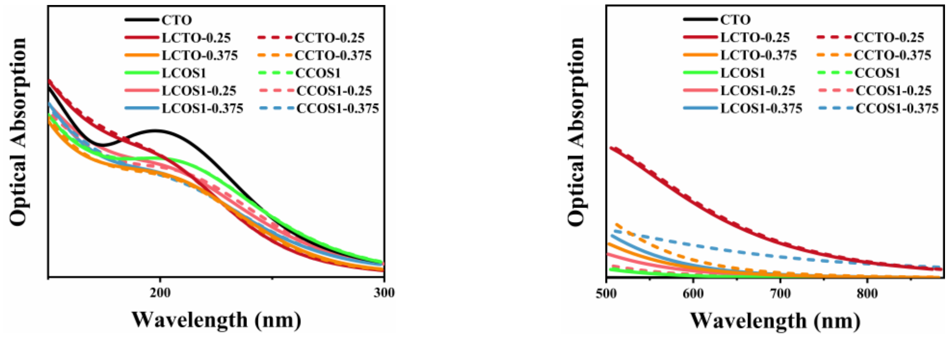

(**a**) (**b**)

Figure S4. Optical absorptions of pure and (La/Ce-N/S) Co-doped CTO: (a) low-wavelength (150-300 nm), (b) high-wavelength (700-900 nm).

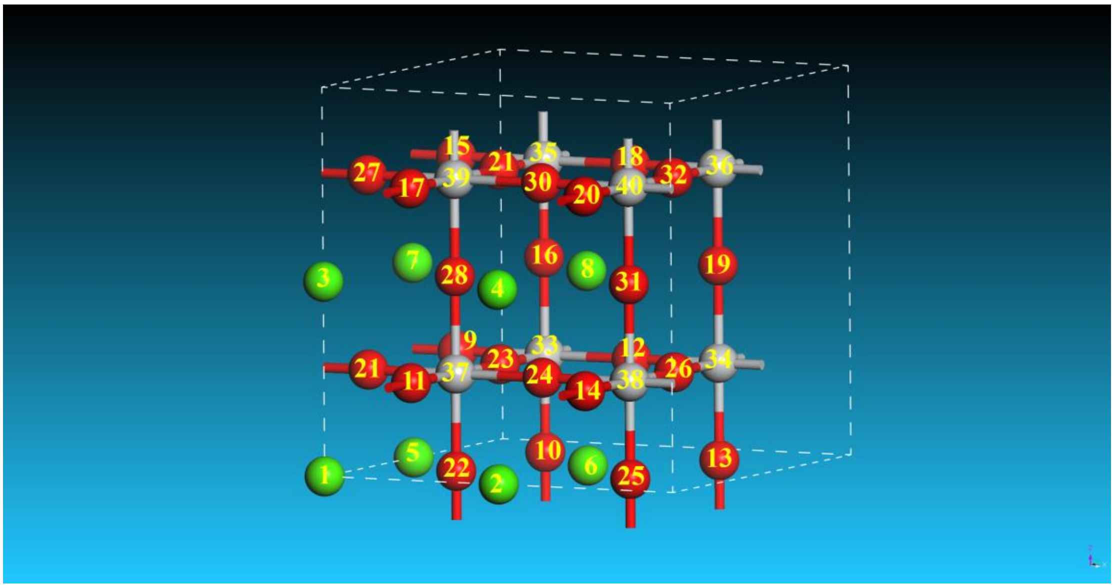

Figure S5. Site indexes for our calculated CTO system.

Table S1. The calculated Bader charge for LCON2 and CCON2.

Model	LCON2		CCON2	
Number	Atom type	Bader net atomic charge	Atom type	Bader net atomic charge
1	Ca	-1.59	Ca	-1.40
2	Ca	-1.63	Ca	-0.89
3	Ca	-1.63	Ca	-1.57
4	Ca	-1.73	Ca	-1.35
5	Ca	-1.58	Ca	-1.44
6	Ca	-1.55	Ca	-1.33
7	Ca	-1.51	Ca	-0.98
8	La	-2.70	Ce	-2.26
9	O	-0.04	O	0.96
10	O	-0.19	O	0.60
11	O	0.77	O	1.11
12	O	1.34	O	0.71
13	O	1.36	O	1.23
14	O	1.34	O	1.28
15	O	1.36	O	0.35
16	O	1.40	O	0.62
17	O	1.35	O	0.95
18	O	1.17	O	0.53
19	O	1.17	O	1.43
20	O	-0.02	O	0.61
21	O	-0.05	O	0.61
22	O	0.79	O	1.28
23	O	0.95	O	1.07
24	O	1.60	O	0.53
25	O	1.16	O	0.96
26	O	1.61	O	1.23
27	N	0.57	N	0.40
28	O	1.14	O	1.38
29	O	1.46	O	0.83
30	O	1.44	O	0.43
31	O	1.08	O	1.22
32	O	1.04	O	0.96
33	Ti	0.08	Ti	-1.04
34	Ti	-1.75	Ti	-0.34
35	Ti	-1.54	Ti	-1.76
36	Ti	-1.70	Ti	-1.51
37	Ti	-1.52	Ti	-1.20
38	Ti	-1.73	Ti	-1.27
39	Ti	-1.74	Ti	-1.28
40	Ti	0.05	Ti	-1.64

Table S2. The calculated Bader charge for LCOS1 and LCOS1-0.375.

**Model**	**LCOS1**		**LCOS1-0.375**	
Number	Atom type	Bader net atomic charge	Atom type	Bader net atomic charge
1	Ca	-1.32	Ca	-1.75
2	Ca	-0.97	La	-1.29
3	Ca	-0.50	Ca	-0.93
4	Ca	-0.70	La	0.63
5	Ca	-0.58	Ca	-0.94
6	Ca	-1.32	Ca	-0.74
7	Ca	-0.77	Ca	-0.36
8	La	-2.41	La	-1.62
9	O	0.80	O	0.93
10	O	0.83	O	1.02
11	O	0.80	O	0.61
12	O	0.97	O	0.89
13	O	0.77	O	0.29
14	O	0.75	O	0.73
15	O	1.07	O	0.71
16	O	1.02	O	0.71
17	O	0.54	O	0.79
18	O	0.76	O	0.61
19	O	0.66	O	0.24
20	O	0.75	O	0.09
21	O	0.98	O	2.01
22	O	0.92	O	1.50
23	O	1.12	O	1.51
24	O	0.94	O	1.78
25	O	0.94	O	1.98
26	O	1.13	O	1.44
27	O	1.94	O	1.38
28	O	0.68	O	0.54
29	O	0.92	O	0.30
30	S	0.05	S	0.50
31	O	0.74	O	0.62
32	O	0.96	O	0.70
33	Ti	-1.78	Ti	-1.98
34	Ti	-1.27	Ti	-1.99
35	Ti	-1.39	Ti	-1.99
36	Ti	-1.54	Ti	-1.69
37	Ti	-1.80	Ti	-1.67
38	Ti	-1.07	Ti	-1.65
39	Ti	-1.26	Ti	-2.01
40	Ti	-1.37	Ti	-1.91

Table S3. The calculated Bader charge for CCOS1, CCOS1-0.25 and CCOS1-0.375.

**Model**	**CCOS1**		**CCOS1-0.25**		**CCOS1-0.375**	
Number	Atom type	Bader net atomic charge	Atom type	Bader net atomic charge	Atom type	Bader net atomic charge
1	Ca	-1.34	Ca	-1.47	Ca	-1.27
2	Ca	-0.79	Ca	-0.38	Ce	-0.86
3	Ca	-0.61	Ca	-0.95	Ca	-0.85
4	Ca	-0.51	Ce	-0.39	Ce	-1.38
5	Ca	-0.83	Ca	-1.38	Ca	-1.21
6	Ca	-0.72	Ca	-0.62	Ca	-0.72
7	Ca	-0.96	Ca	-1.11	Ca	-0.40
8	Ce	-1.77	Ce	-0.84	Ce	-1.00
9	O	0.69	O	0.57	O	1.11
10	O	0.95	O	1.02	O	0.88
11	O	0.50	O	0.91	O	0.88
12	O	0.93	O	0.94	O	1.31
13	O	0.71	O	0.92	O	0.75
14	O	0.60	O	0.75	O	0.96
15	O	0.94	O	0.78	O	0.83
16	O	1.19	O	1.23	O	0.98
17	O	0.63	O	0.45	O	0.71
18	O	0.89	O	1.24	O	1.29
19	O	1.11	O	1.11	O	1.17
20	O	0.87	O	0.11	O	0.44
21	O	0.73	O	0.78	O	1.17
22	O	0.64	O	0.84	O	0.69
23	O	0.64	O	0.31	O	0.20
24	O	1.07	O	1.15	O	1.24
25	O	0.81	O	0.92	O	0.92
26	O	0.64	O	0.60	O	0.38
27	O	1.96	O	1.60	O	1.73
28	O	1.10	O	1.08	O	1.07
29	O	0.67	O	0.98	O	0.46
30	S	0.19	S	0.55	S	0.49
31	O	0.97	O	1.47	O	1.24
32	O	0.66	O	0.91	O	0.60
33	Ti	-1.54	Ti	-1.63	Ti	-1.95
34	Ti	-1.67	Ti	-1.78	Ti	-1.74
35	Ti	-1.10	Ti	-1.01	Ti	-1.13
36	Ti	-1.52	Ti	-1.86	Ti	-1.75
37	Ti	-1.96	Ti	-1.92	Ti	-1.93
38	Ti	-1.22	Ti	-1.85	Ti	-1.72
39	Ti	-1.62	Ti	-1.42	Ti	-1.31
40	Ti	-0.94	Ti	-1.58	Ti	-1.30
